# Immunisation with Recombinant PfEMP1 Domains Elicits Functional Rosette-Inhibiting and Phagocytosis-Inducing Antibodies to *Plasmodium falciparum*

**DOI:** 10.1371/journal.pone.0016414

**Published:** 2011-01-31

**Authors:** Ashfaq Ghumra, Pongsak Khunrae, Ricardo Ataide, Ahmed Raza, Stephen J. Rogerson, Matthew K. Higgins, J. Alexandra Rowe

**Affiliations:** 1 Centre for Immunity, Infection and Evolution, Institute of Immunology and Infection Research, School of Biological Sciences, University of Edinburgh, Edinburgh, United Kingdom; 2 Department of Biochemistry, University of Cambridge, Cambridge, United Kingdom; 3 Department of Medicine, University of Melbourne, Post Office Royal Melbourne Hospital, Melbourne, Australia; 4 Graduate Program in Areas of Basic and Applied Biology, Universidade do Porto, Porto, Portugal; Université Pierre et Marie Curie, France

## Abstract

**Background:**

Rosetting is a *Plasmodium falciparum* virulence factor implicated in the pathogenesis of life-threatening malaria. Rosetting occurs when parasite–derived *P. falciparum* Erythrocyte Membrane Protein One (PfEMP1) on the surface of infected erythrocytes binds to human receptors on uninfected erythrocytes. PfEMP1 is a possible target for a vaccine to induce antibodies to inhibit rosetting and prevent severe malaria.

**Methodology/Findings:**

We examined the vaccine potential of the six extracellular domains of a rosette-mediating PfEMP1 variant (ITvar9/R29var1 from the R29 parasite strain) by immunizing rabbits with recombinant proteins expressed in *E. coli*. Antibodies raised to each domain were tested for surface fluorescence with live infected erythrocytes, rosette inhibition and phagocytosis-induction. Antibodies to all PfEMP1 domains recognized the surface of live infected erythrocytes down to low concentrations (0.02–1.56 µg/ml of total IgG). Antibodies to all PfEMP1 domains except for the second Duffy-Binding-Like region inhibited rosetting (50% inhibitory concentration 0.04–4 µg/ml) and were able to opsonize and induce phagocytosis of infected erythrocytes at low concentrations (1.56–6.25 µg/ml). Antibodies to the N-terminal region (NTS-DBL1α) were the most effective in all assays. All antibodies were specific for the R29 parasite strain, and showed no functional activity against five other rosetting strains.

**Conclusions/Significance:**

These results are encouraging for vaccine development as they show that potent antibodies can be generated to recombinant PfEMP1 domains that will inhibit rosetting and induce phagocytosis of infected erythrocytes. However, further work is needed on rosetting mechanisms and cross-reactivity in field isolates to define a set of PfEMP1 variants that could induce functional antibodies against a broad range of *P. falciparum* rosetting parasites.

## Introduction

The global mortality from malaria continues to be a huge problem, with *Plasmodium falciparum* being the major cause of severe, life-threatening malaria in African children [1]. Rosetting, the binding of infected erythrocytes to two or more uninfected erythrocytes, has been shown to be one of the main parasite virulence phenotypes associated with severe malaria. Initial studies showed high levels of rosetting in parasite isolates from patients with cerebral malaria [2], [3], with subsequent work showing rosetting to be linked to all forms of severe malaria [4], [5], [6], [7], [8]. Results from human genetic studies have shown that erythrocyte polymorphisms that reduce rosetting (complement receptor 1 deficiency [9] and blood group O [5]), confer protection against severe malaria, reducing the odds ratio for severe disease by about two thirds [10], [11]. This protective effect may occur because these polymorphisms reduce the vaso-occlusive effects of rosetting [12], thought to be a key pathological process in severe malaria [13]. Together, the association of rosetting with severe malaria, and the protective effect of human rosette-reducing polymorphisms, supports a direct role for rosetting in the pathogenesis of severe malaria. Therapeutic interventions that target rosetting may therefore have potential to decrease the global burden of severe malaria [14], [15]. This is further supported by the observation that rosette-inhibiting antibody responses are associated with protection from severe malaria [2].

Rosetting is mediated by *P. falciparum* Erythrocyte Membrane Protein-1 (PfEMP1) expressed on the surface of mature infected erythrocytes [9]. PfEMP1 variants are 200–400 kDa proteins encoded by a repertoire of ∼60 *var* genes per haploid parasite genome, and consisting of tandemly arranged Duffy Binding Like (DBL) and Cysteine-rich InterDomain Region (CIDR) domains [16]. *Var* genes can be classified into groups A, B and C according to their 5′ non-coding sequences, chromosomal location and gene orientation [16]. Existing data on *var* gene groups and rosetting are not entirely consistent. Two well-characterized rosette-mediating variants are encoded by Group A *var* genes (*ITvar9*, also known as *R29var1* [9], and *varO* [17]), while a third putative rosette-mediating variant (encoded by *FCR3S1.2var1*) is group B or C [18]. In *P. falciparum* field isolates, there is a strong positive correlation between group A *var* gene transcription and parasite rosette frequency [19], [20], [21], [22], suggesting that group A PfEMP1 variants are common rosetting ligands in natural populations.

Currently, there are few data on the vaccine potential of rosette-mediating PfEMP1 variants. Previous work has shown that the N-terminal DBL1α domain is the functional erythrocyte binding region of rosette-mediating PfEMP1 variants [9], [17], [23], making this domain the most promising candidate for an anti-rosetting vaccine. Antibodies to DBL1α of the VarO variant from the Palo Alto parasite strain are effective at disrupting rosettes [50% Inhibitory Concentration (IC50) against Palo Alto, approximately 1/200 dilution of serum [17]], while antibodies to the DBL1α domain of the FCR3S1.2var1 variant have only a modest effect (IC50 against FCR3S1.2 parasites at 1/2 dilution of serum) [24]. As stated above, *FCR3S1.2var1* is a group B or C *var* gene, and the majority of the other data suggest that rosetting and severe malaria are associated with group A *var* genes [19], [20], [21], [22]. Therefore the relevance of *FCR3S1.2var1* is unclear, and rosette-mediating group A variants may be better suited for preliminary studies on the potential for anti-rosetting vaccines.

It remains unclear whether only DBL1α can induce rosette-disrupting antibodies, or whether the other DBL and CIDR domains from rosette-mediating PfEMP1 variants can also generate effective anti-rosetting activity. In addition, it is unknown whether distinct DBL and CIDR domains differ in their ability to induce cross-reactive antibodies that are effective against multiple parasite strains. Finally, the ability of antibodies to recombinant PfEMP1 domains to promote clearance of infected erythrocytes via opsonization and phagocytosis, which would also be desirable in a vaccine, has not previously been studied. We therefore expressed all of the extracellular domains from a rosette-mediating group A PfEMP1 variant (ITvar9/R29var1) as recombinant proteins in *E. coli*, in order to investigate which domains of PfEMP1 elicit rosette-inhibiting and phagocytosis-inducing antibodies, and to determine the cross-reactivity of the antibodies for other rosetting parasite strains.

## Results

### Expression of DBL and CIDR domains from ITvar9 as recombinant proteins in *E. coli*

**Figure 1:**
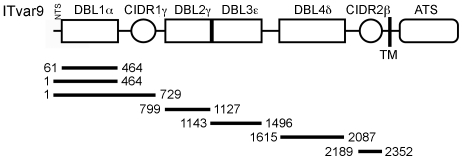
Diagram of the ITvar9 PfEMP1 variant showing domains expressed as recombinant proteins in *E.coli*. The *ITvar9* gene (also known as *R29var1* [9], accession numbers Y13402 and CAA73831) encodes a group A PfEMP1 variant with four Duffy Binding Like (DBL) and two Cysteine-rich InterDomain Region (CIDR) domains. The number of the domain designates its position from the N-terminus and the Greek symbol represents its homology group [64]. PfEMP1 contains a N-Terminal Segment (NTS) and an Acidic Terminal Segment (ATS) proximal to the transmembrane (TM) region. The DBL1α domain is the functional erythrocyte-binding region [9]. The bars represent the *E.coli* expression constructs and the numbers show the first and last amino acid positions.

**Figure 2:**
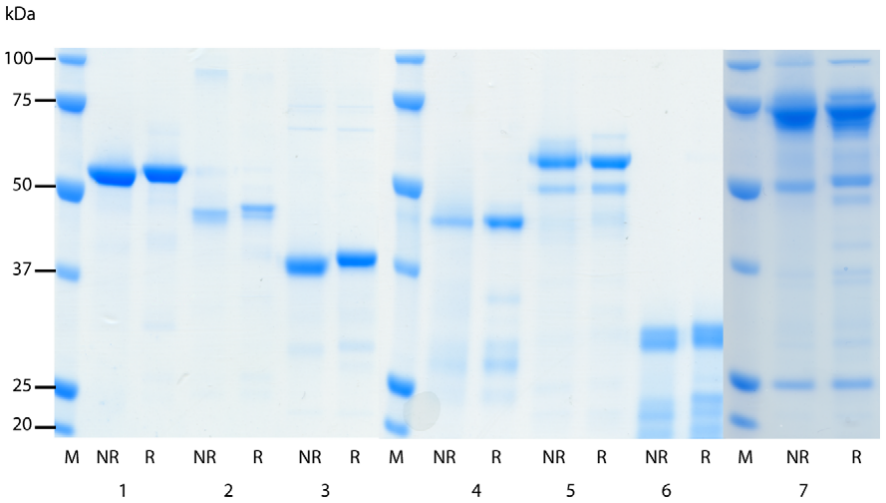
SDS-PAGE showing recombinant DBL and CIDR domains from ITvar9 expressed in *E. coli*. The purity and quality of the recombinant DBL and CIDR domains were assessed by electrophoresis of reduced and non-reduced pairs of proteins on 10% SDS-polyacrylamide gels. Two µg of protein was used per well and lanes were as follows: 1) NTS-DBL1α, 2) DBL1α, 3) DBL2γ 4) DBL3ε, 5) DBL4δ, 6) CIDR2β and 7) NTS-DBL1α-CIDR1γ. M, molecular weight marker; NR, non-reduced; R, reduced.

Domains from the group A rosette-mediating PfEMP1 variant ITvar9 (also known as R29var1 [9]) were expressed as His-tagged proteins in *E.coli* (Figure 1). Previous difficulties in expressing PfEMP1 domains in *E.coli* [17], [24], [25], [26], [27], [28] were overcome using the methods developed by Higgins [29]. DBL and CIDR structural information [30], [31], [32] was used to guide the design of domain boundaries, and proteins were expressed using a modified pET15b vector in Origami B cells, supplemented with the pRIG vector [33] to enrich for rare tRNAs [29], [34]. All ITvar9 PfEMP1 extracellular domains were expressed individually, except for the first CIDR, which could only be expressed successfully as part of the NTS-DBL1α-CIDR1γ di-domain (Figure 1). The recombinant proteins were all soluble except for DBL1α, which occurred in insoluble inclusion bodies and was refolded as described in the methods. Addition of the short N-terminal sequence (NTS) to DBL1α resulted in production of soluble protein, suggesting that the NTS may be an integral part of the DBL1α domain. After purification and removal of the His-tag, two µg of each recombinant protein was electrophoresed on an SDS-polyacrylamide gel under reducing and non-reducing conditions. All proteins showed a major band at the expected molecular weight, with a variable amount of smaller degradation products (Figure 2). All proteins except for DBL4δ showed a band shift in the presence of β-mercaptoethanol, indicating the reduction of disulphide bonds (Figure 2). Protein yields ranged from 2mg/l for NTS-DBL1α down to 0.1mg/l for CIDR2β and DBL3ε.

### Immunisation of rabbits to produce polyclonal antibodies that recognize native PfEMP1 on the surface of R29 live infected erythrocytes

Each recombinant protein shown in Figure 2 was used to immunise two rabbits. The antisera showed good recognition of the proteins used for immunisation by ELISA, with 50% titres (defined as the titre giving 50% of the maximum OD) ranging between 1/40,000 (DBL4δ) and 1/500,000 (NTS-DBL1α, see footnote to Table 1 for the full list of titres). The two rabbits used for each immunogen showed only minor differences in ELISA titre (data not shown).

**Table 1:** Effectiveness of ITvar9 antibodies in various assays#.

Antibodies	IFA end titre$ (µg/ml)	Rosette Inhibition IC50* (µg/ml)	Phagocytosis ∼50% of positive control (µg/ml)
Negative control rabbit IgG	Negative at 100	Negative at 500	Not done
Anti-NTS-DBL1α	**0.024** †	**0.04**	**<1.56**
Anti-DBL1α	0.39	0.1	6.25
Anti-NTS-DBL1α-CIDR1γ	0.10	0.05	1.56
Anti-DBL2γ	0.39	>500	100
Anti-DBL3ε	0.10	2	1.56
Anti-DBL4δ	0.10	1	6.25
Anti-CIDR2β	1.56	4	6.25

# 50% ELISA titres for the antibodies (defined as the titre giving 50% of the maximum OD) were as follows: NTS-DBL1α 1/500,000; DBL1α 1/250,000; NTS-DBL1α-CIDR1γ 1/300,000; DBL2γ 1/50,000; DBL3ε 1/200,000; DBL4δ 1/40,000 and CIDR2β 1/200,000.

$ The lowest concentration at which >50% of the infected erythrocytes in the culture showed punctate fluorescence by IFA. Values shown are µg/ml of purified total IgG.

*50% inhibitory concentration (IC50) for rosette inhibition. Values shown are µg/ml of purified total IgG.

† The most effective antibodies in each assay are shown in bold. Values shown are µg/ml of purified total IgG.

**Figure 3:**
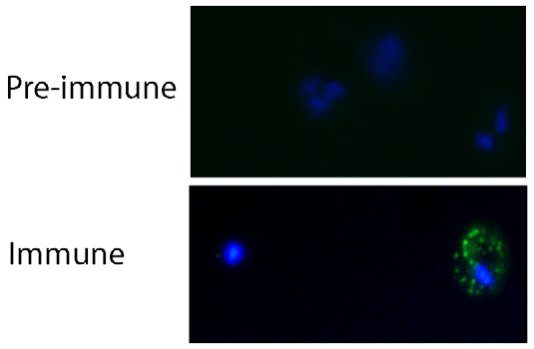
Immunofluorescence assay showing that ITvar9 antibodies recognize PfEMP1 on the surface of live infected erythrocytes. R29 mature infected erythrocytes (pigmented trophozoites and schizonts) were grown to 5% parasitaemia and incubated with rabbit antisera against recombinant ITvar9 DBL and CIDR domains at 1/50 dilution. After washing, the cells were incubated with Alexa Fluor 488-labelled goat anti-rabbit IgG (Invitrogen) at 1/1000 dilution. The example shown here is the binding of anti-DBL2γ antisera, however all antisera to ITvar9 gave similar results. Punctate staining of the membrane of infected erythrocytes (green) was seen with the specific antisera (“immune”) but not with the pre-immune sera. The location of infected erythrocytes is shown by DAPI staining of the parasite (blue). Slides were viewed with a 100× objective using a Leica DM 2000 fluorescent microscope.

Immunofluorescence assays (IFA) were carried out to test whether the rabbit antisera at 1/50 dilution contained antibodies that recognized native PfEMP1 on the surface of R29 live infected erythrocytes. For every recombinant protein, antisera from both rabbits showed binding to R29 infected erythrocytes, whereas no binding was detected with the pre-immune sera (Figure 3). For all antisera, a punctate fluorescent pattern was observed across the surface of the infected erythrocyte membrane, similar to that seen previously for PfEMP1-binding antibodies [35], [36], [37] (Figure 3). The percentage of infected erythrocytes showing a positive fluorescence signal closely matched the rosette frequency of the culture (55–70%), suggesting that the antibodies specifically recognized the rosetting infected erythrocytes. Although bright punctate fluorescence on infected erythrocytes was seen with all antisera, the intensity of the fluorescence staining was often stronger for one rabbit out of the pair immunised with each protein.

As a further negative control, an IFA was carried out with antibodies to DBL1α domains from several other PfEMP1 variants (*ITvar60*, *HB3var3*, *HB3var6*, *TM180var1*, *TM284var1 and Muz12var1*, Ghumra and Rowe, in preparation). These control antibodies were negative with R29 parasites, however they do recognize live infected erythrocytes of the parasite strain in which they are predominantly expressed (Ghumra and Rowe, in preparation). This indicates that DBL antibodies in general do not recognize R29, only specific antibodies to ITvar9, the predominantly expressed variant in R29 parasites [9].

For each ITvar9 recombinant protein, the antiserum giving the brightest signal by IFA was chosen for purification of total IgG, for use in rosetting and phagocytosis assays below. In order to compare the relative abilities of the purified IgG preparations to recognize live infected erythrocytes, we titred out the IFA signal. This was done by incubating parasite culture suspension with IgG at four-fold dilutions, starting at 25 µg/ml then 6.25, 1.56, 0.39, 0.01, 0.024, 0.006 and 0.0015 µg/ml. The end titre was defined here as the lowest concentration at which >50% of the infected erythrocytes in the culture showed punctate fluorescence. Although IFA results can be considered subjective, the end titres in these experiments were clear, as demonstrated by two independent observers identifying the same end titre in all cases. The end titres for positive punctate fluorescence on live R29 infected erythrocytes ranged from 0.024 µg/ml for anti-NTS-DBL1α to 1.56 µg/ml for anti-CIDR2β (Table 1). Antibodies to all domains except CIDR2β showed agglutination of infected erythrocytes at 25 µg/ml (mostly small 5–10 cell agglutinates under these conditions). Agglutinates did not occur at lower concentrations.

### Antibodies to NTS-DBL1α of ITvar9 disrupt existing rosettes and inhibit formation of new rosettes

To study whether antibodies to specific PfEMP1 domains interfere with rosetting, we carried out two types of assay. Rosette disruption assays use mature pigmented trophozoites and determine whether antibodies can reverse pre-existing rosettes [38]. Rosette inhibition assays determine whether antibodies added to parasite culture at ring stage (before rosetting begins) and incubated overnight, prevent the formation of rosettes when the parasites mature to the pigmented trophozoite stage. The latter assay may be more sensitive [38], whereas the former is technically quicker and easier.

**Figure 4:**
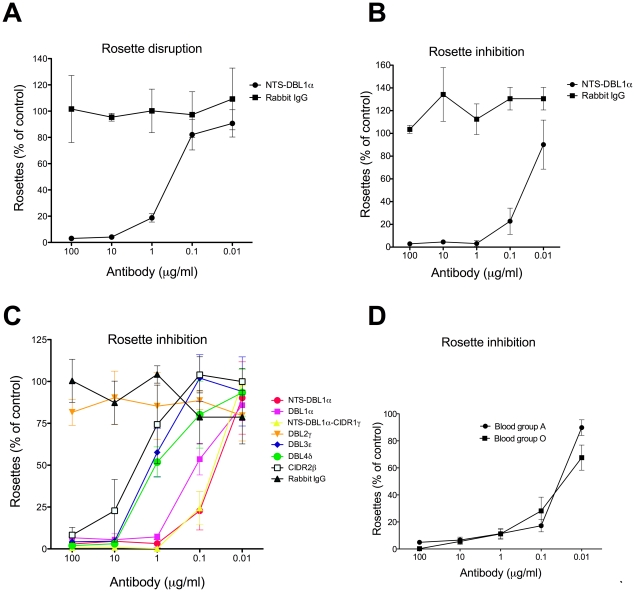
Antibodies to ITvar9 domains have anti-rosetting activity against R29 parasites. Purified IgG raised against NTS-DBL1α disrupted existing rosettes (A) and inhibited the formation of rosettes (B), whereas negative control non-immunized rabbit IgG had no effect on rosetting (A and B). Antibodies raised to all extracellular domains of the ITvar9 PfEMP1 variant inhibited R29 rosetting except for DBL2γ antibodies (C). NTS-DBL1α antibodies showed similar dose-response curves when the R29 parasites were grown in group A or O erythrocytes (D). The results are expressed as percentage of the control value, in which complete binding medium (equivalent volume as that of antibody) was added to the parasite culture. The control always had at least 50% of infected erythrocytes in rosettes. For (A), the mean and standard deviation of three independent experiments is shown. For (B), (C) and (D), the mean and standard deviation of triplicate determinations of rosette frequency at each concentration of antibody within a single experiment is shown.

In the first instance, the rosette-disrupting ability of antibodies against the NTS-DBL1α of ITvar9 was examined with R29 parasites. The antibodies were tested at a range of concentrations from 0.01 to 100 µg/ml of total IgG. We found that 10 µg/ml of NTS-DBL1α antibody could abolish rosetting completely, with a 50% inhibitory concentration (IC50) value for rosette disruption of 0.4 µg/ml (Figure 4A). Negative control non-immunised rabbit IgG did not disrupt R29 rosettes (Figure 4A).

The NTS-DBL1α antibodies were also effective in rosette inhibition assays, giving complete inhibition of R29 rosetting at 1 µg/ml, and an IC50 value of 0.04 µg/ml (Figure 4B). Therefore a lower concentration of antibody was required to inhibit rosette formation than to break up pre-existing rosettes. Negative control non-immunised rabbit IgG did not inhibit the formation of R29 rosettes (Figure 4B). Parasite growth and maturation in the presence of the antibodies was normal as assessed by Giemsa smear. Due to the greater sensitivity of the rosette inhibition assay, this was used in subsequent experiments.

Antibodies to the other recombinant proteins containing DBL1α (DBL1α alone, and the di-domain of NTS-DBL1α-CIDR1γ) were also tested in rosette inhibition assays. These antibodies showed very similar anti-rosetting activity to that shown above for NTS-DBL1α, with almost complete inhibition of rosetting at 1 µg/ml, and IC50 values of approximately 0.05–0.1 µg/ml) (Figure 4C).

### Antibodies to other domains of ITvar9 also inhibit rosette formation

Antibodies against the four other extracellular domains of ITvar9 (Figure 1) were used in rosette inhibition assays and showed a varying degree of anti-rosetting activity (Figure 4C). Anti-DBL3ε, anti-DBL4δ and anti-CIDR2β were all able to abolish rosetting, although higher concentrations were needed than for anti-DBL1α antibodies. The approximate IC50 for rosette inhibition of anti-DBL3ε and anti-DBL4δ was 1–2 µg/ml, and 4 µg/ml for anti-CIDR2β (Figure 4C). Anti-DBL2γ showed no rosette inhibition over the range of concentrations shown in Figure 4C, but did show modest rosette inhibition at higher concentrations (≥500 µg/ml). As with the previous assays, negative control rabbit IgG did not inhibit rosetting at equivalent concentrations, and parasite growth and maturation were not affected by any of the antibodies.

Therefore, antibodies to multiple domains of the rosette-mediating ITvar9 variant were able to inhibit rosette formation of R29 infected erythrocytes, although antibodies to recombinant proteins containing DBL1α were the most effective (Figure 4 and Table 1). Antibodies to DBL2γ only showed modest effects at very high concentrations.

The above experiments were all carried out with R29 parasites grown in group O erythrocytes. We also assessed the effect of NTS-DBL1α antibodies against parasites grown in group A erythrocytes, in which R29 forms larger rosettes than in O cells (although overall rosette frequency is not affected). The NTS-DBL1α antibodies were equally effective against R29 parasites whether in group O or group A erythrocytes (Fig 4D).

### Antibodies to non-DBL1α domains that inhibit rosetting do so without showing cross-reactivity with DBL1α

**Figure 5 pone-0016414-g005:**
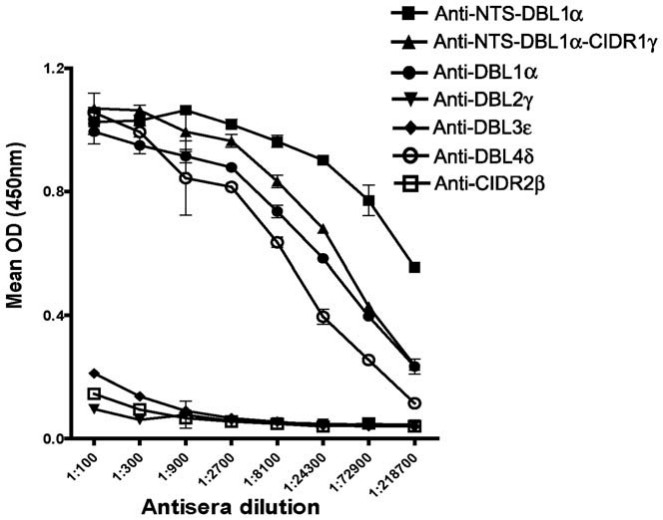
ELISA to detect binding of PfEMP1 antibodies to recombinant NTS-DBL1α. Antibodies were added to wells coated with 2 µg/ml of recombinant NTS-DBL1α protein and binding was detected using HRP-conjugated anti-rabbit IgG at 1/10,000 dilution. Antisera raised to recombinant proteins containing DBL1α (i.e. anti-NTS-DBL1α, anti-DBL1α and anti-NTS-DBL1α-CIDR1γ) all recognize the recombinant protein as expected. Antisera to DBL2γ, DBL3ε and CIDR2β do not cross-react with recombinant NTS-DBL1α. However, the antiserum to DBL4δ does shows binding to the recombinant NTS-DBL1α, suggesting that there is cross-reactivity between these two domains.

DBL1α has previously been identified as the erythrocyte-binding domain from ITvar9 [9], therefore it was expected that antibodies to DBL1α would inhibit rosetting. However, it was surprising to discover that antibodies to all the other extracellular domains of the ITvar9 variant could also inhibit rosetting. We considered the possibility that the antibodies to the other domains might affect rosetting due to cross-reactivity with DBL1α. To investigate this, we carried out an ELISA to determine the ability of each anti-PfEMP1 IgG to recognize the NTS-DBL1α recombinant protein. As expected, the NTS-DBL1α protein was well-recognized by the antibodies raised to recombinant proteins containing DBL1α (i.e. anti-NTS-DBL1α, anti-DBL1α and anti-NTS-DBL1α-CIDR1γ) (Figure 5). The antibodies to DBL2γ, DBL3ε and CIDR2β did not recognize NTS-DBL1α, suggesting that the rosette-inhibiting activity of these antibodies is not likely to be due to cross-reactivity with DBL1α. The DBL4δ antibodies did, however, bind well to NTS-DBL1α protein, raising the possibility that rosette inhibition of anti-DBL4δ could be due to cross-reactivity. The DBL4δ antibodies bound to DBL4δ protein at a higher ELISA titre (50% of maximum OD at approximately 1/40,000, data not shown) than the NTS-DBL1α protein (50% of maximum OD at approximately 1/10,000, Figure 5). This indicates that the DBL4δ antibodies show good recognition of the specific immunising protein (DBL4δ), and that the reactivity with NTS-DBL1α protein is not due to sample mix-up and immunisation with the wrong protein.

**Figure 6:**
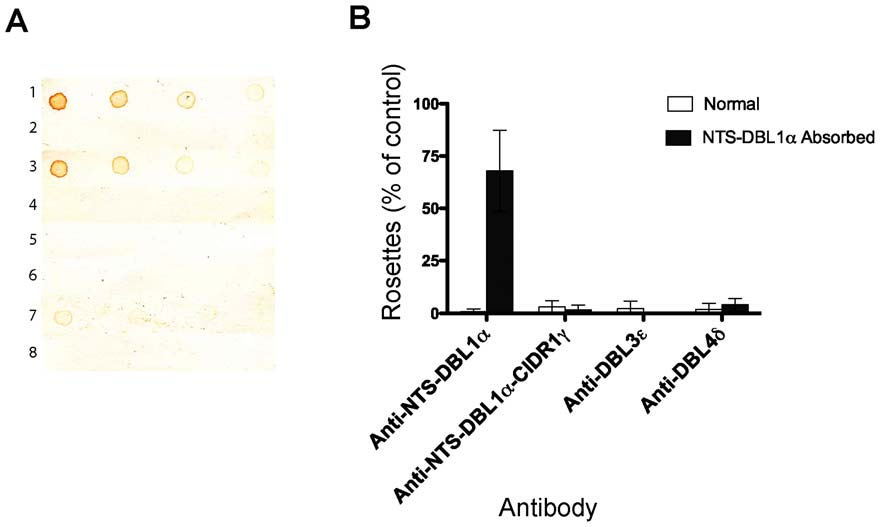
Rosette inhibition of antibodies depleted by absorption against NTS-DBL1α. Immunoblotting (A) and rosette inhibition (B) by pairs of antibodies that were either non-absorbed, or absorbed on NTS-DBL1α recombinant protein coupled to sepharose. A) Recombinant NTS-DBL1α protein was spotted onto nitrocellulose membrane at doubling dilutions, starting from 2 µg/ml, and incubated with 1/1000 dilution of absorbed or non-absorbed antibody. 1) non-absorbed anti-NTS-DBL1α, 2) absorbed anti-NTS-DBL1α, 3) non-absorbed anti-NTS-DBL1α-CIDR1γ, 4) absorbed anti-NTS-DBL1α-CIDR1γ, 5) non-absorbed anti-DBL3ε, 6) absorbed anti-DBL3ε, 7) non-absorbed anti-DBL4δ and 8) absorbed anti-DBL4δ. Non-absorbed antibodies to DBLα (lanes 1 and 3) and DBL4δ (lane7) recognized NTS-DBL1α recombinant protein. After absorption, however, this activity was lost (lanes 2, 4 and 8). Antibodies to DBL3ε did not recognize NTS-DBL1α recombinant protein (lanes 4 and 5). B) Rosette inhibition assays showed that the anti-rosetting activity of NTS-DBL1α antibodies was lost after absorption. Antibodies to DBL3ε and DBL4δ retained rosette-inhibitory activity after absorption, showing that their anti-rosetting effects are likely to be independent of DBL1α. Antibodies to NTS-DBL1α-CIDR1γ also retained inhibitory effects after absorption on NTS-DBL1α protein, suggesting that antibodies to the CIDR1γ domain of ITvar9 also have anti-rosetting effects. Data shown are the mean and standard deviation of triplicate determinations of rosette frequency after overnight incubation with absorbed or non-absorbed antibody diluted 1/10 from the 1 mg/ml stock used for absorption. The control (with binding medium only added) had more than 50% of infected erythrocytes in rosettes.

To test whether anti-DBL4δ did inhibit rosetting through binding to DBL1α, we carried out a pre-absorption experiment using NTS-DBL1α protein coupled to sepharose beads. Firstly, IgG (anti-NTS-DBL1α, anti-NTS-DBL1α-CIDR1γ, anti-DBL3ε and anti-DBL4δ) were pre-absorbed against NTS-DBL1α-sepharose and then used to detect recombinant NTS-DBL1α, spotted onto nitrocellulose. Before absorption, antisera to NTS-DBL1α, NTS-DBL1α-CIDR1 and DBL4δ bound to NTS-DBL1α protein, but after absorption this activity was lost, indicating removal of the anti-NTS-DBL1α IgG (Figure 6A, tracks 1–4 and 7–8). As expected from the ELISA (Figure 5), immunoblotting confirmed that unabsorbed anti-DBL3ε did not bind NTS-DBL1α (Figure 6A, track 5), and these antibodies were therefore used in the subsequent rosette inhibition assays to control for any non-specific effects of the absorption process.

Rosette inhibition was carried out with absorbed and non-absorbed antibodies. The positive control, anti-NTS-DBL1α, showed that rosette-inhibition was lost after pre-absorption on NTS-DBL1α protein (Figure 6B). The negative control, anti-DBL3ε, abolished rosetting both before and after absorption on NTS-DBL1α protein, indicating that the absorption process itself does not non-specifically remove anti-rosetting activity. The anti-DBL4δ also showed equally effective rosette inhibition before and after absorption, indicating that the rosette-inhibiting activity of anti-DBL4δ antibodies is not due to cross-reactivity with DBL1α (Figure 6B).

### Antibodies to CIDR1γ of ITvar9 also inhibit rosetting

The only region of ITvar9 that we were unable to express as a single domain was CIDR1γ, although we were able to express it as part of a di-domain with DBL1α (Figures 1 and 2). As shown in Figure 4, antibodies to the di-domain effectively inhibit rosetting, however, this could be solely due to antibodies to the DBL1α component. It is not possible to determine from Figure 4 whether antibodies to the CIDR1γ domain also have anti-rosetting activity. We therefore used the pre-absorption experiment described above to investigate the effect of removing antibodies to NTS-DBL1α from the di-domain antisera. We found that pre-absorbed anti-NTS-DBL1α-CIDR1 maintained its rosette inhibiting activity (Figure 6B) suggesting that antibodies binding to epitopes in CIDR1γ can also inhibit rosetting. It should be noted that a limitation of this experiment is that it is unknown at present whether NTS-DBL1α structure within a didomain is identical to NTS-DBL1α alone. It is possible that different DBL1α epitopes are exposed in the didomain, and antibodies to these epitopes would not be removed by absorption on NTS-DBL1α alone. This possibility cannot be excluded at present, and will only be resolved when further structural information on the PfEMP1 NTS-didomain is available.

Taken together, the data in Figures 4–6 show that antibodies can be targeted to multiple domains of PfEMP1 to achieve rosette inhibition.

### Antibodies to ITvar9 are strain-specific

**Figure 7:**
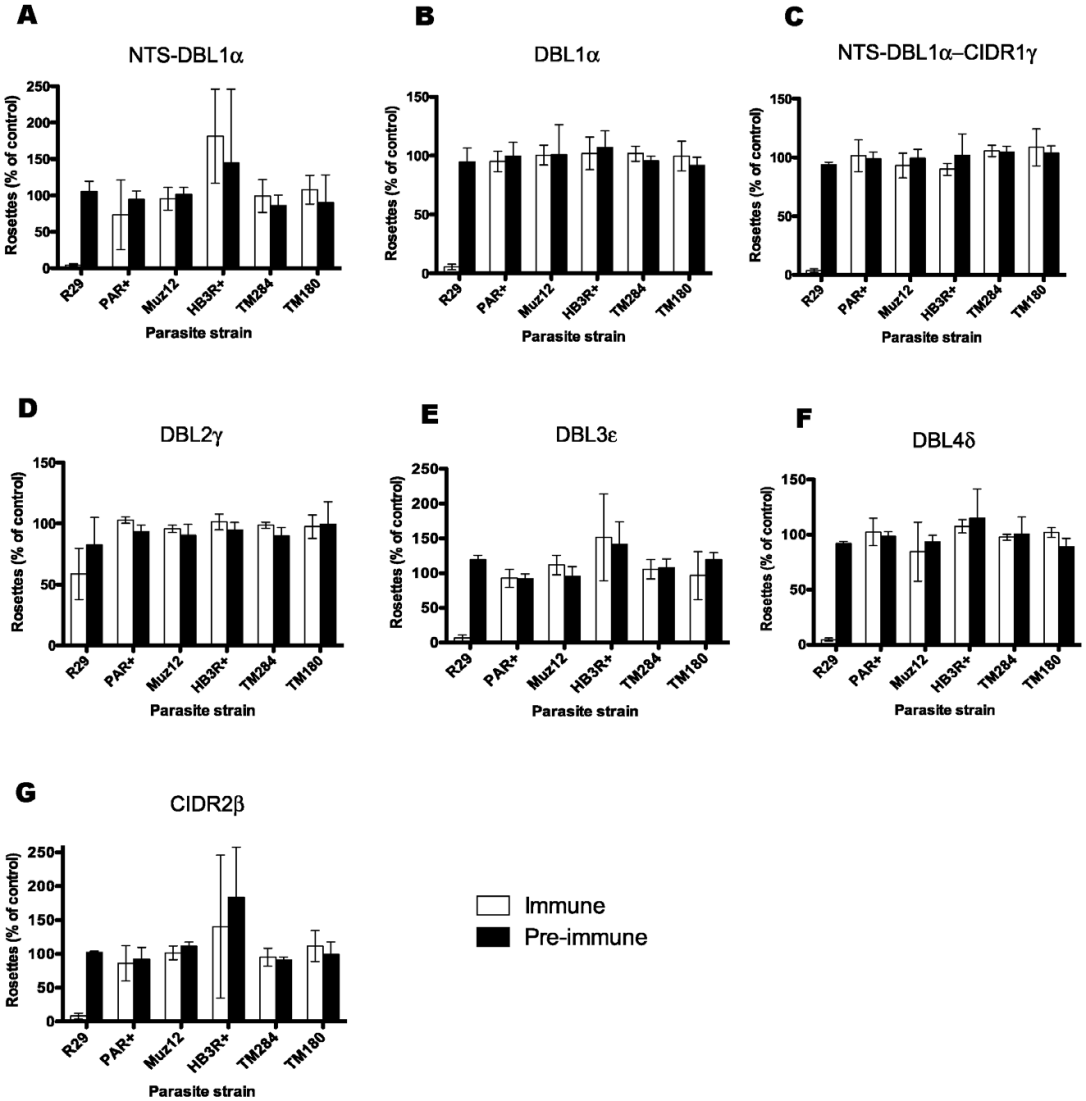
Rosette disruption assays with ITvar9 antisera against six *P. falciparum* rosetting laboratory strains. Antisera raised to ITvar9 domains, and paired pre-immune sera, were used at 1/20 dilution in rosette disruption assays with R29, PAR+, Muz12, HB3R+, TM284 and TM180. Antisera were as follows: A) NTS-DBL1α, B) DBL1α, C) NTS-DBL1α-CIDR1γ, D) DBL2γ, E) DBL3ε, F) DBL4δ and G) CIDR2β. Disruption of rosetting was only seen with R29 parasites. Data shown are the mean and standard deviation from three independent experiments. The control (with binding medium only added) had more than 50% of infected erythrocytes in rosettes.

**Figure 8:**
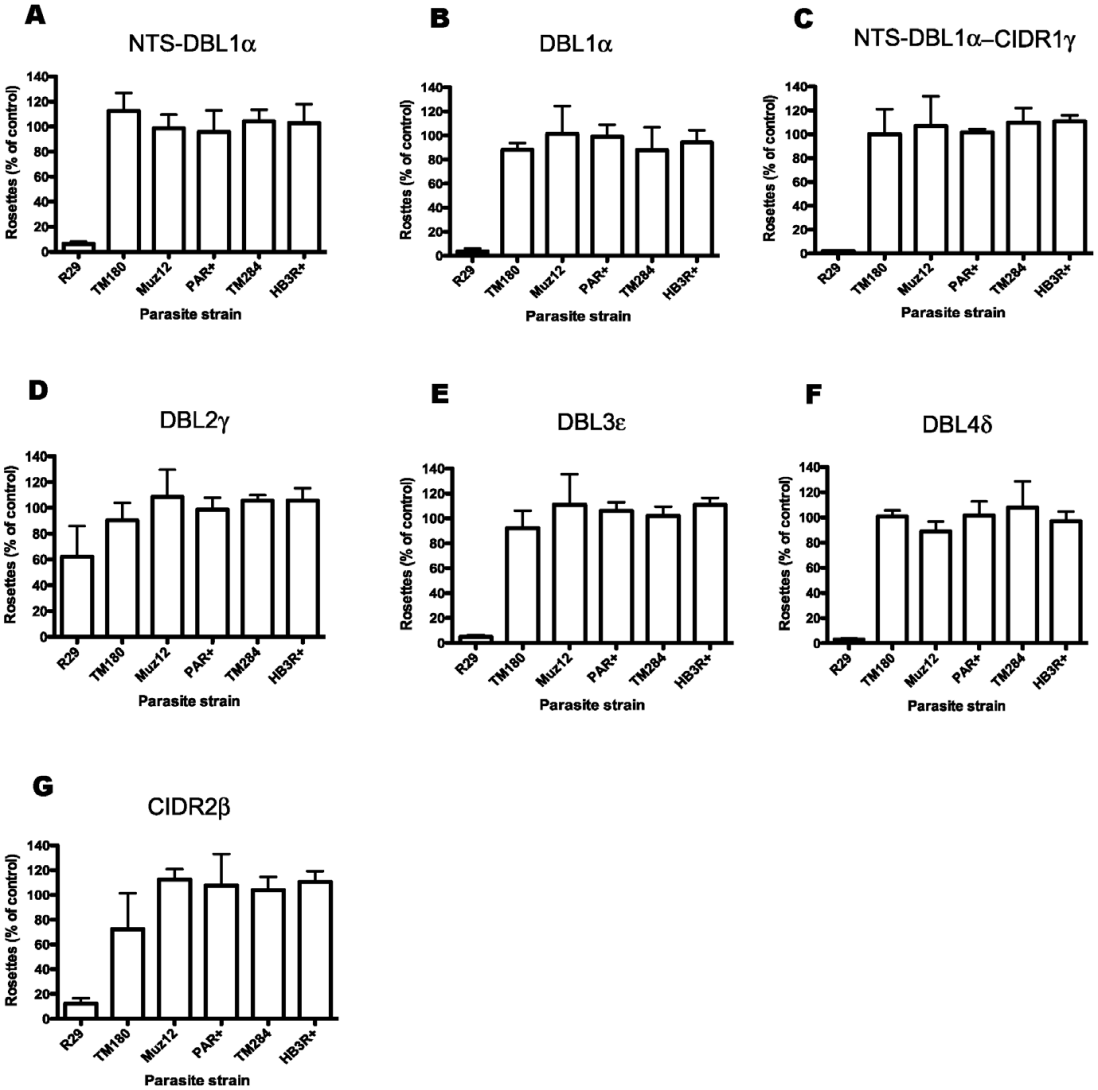
Rosette inhibition assays with ITvar9 antisera against six *P. falciparum* rosetting laboratory strains. Total IgG was used at a concentration of 100 µg/ml in rosette inhibition assays with R29, PAR+, Muz12, HB3R+, TM284 and TM180. Antisera were as follows: A) NTS-DBL1α, B) DBL1α, C) NTS-DBL1α-CIDR1γ, D) DBL2γ, E) DBL3ε, F) DBL4δ and G) CIDR2β. Inhibition of rosetting was only seen with R29 parasites. Data shown are the mean and standard deviation of triplicate determinations of rosette frequency within a single experiment. The control (with binding medium only added) had more than 50% of infected erythrocytes in rosettes.

To investigate the antigenic relationship of different rosette-mediating PfEMP1 variants, the rabbit antibodies against the ITvar9 domains were tested in IFA and rosetting assays against five different *P. falciparum* rosetting laboratory strains: PAR+, TM284, Muz12, HB3R+ and TM180. The PfEMP1 variants from these strains show between 28 and 35% amino acid identity with ITvar9 over the extracellular region (Ghumra and Rowe, in preparation). All of the antibodies to ITvar9 domains at 100 µg/ml were negative by IFA with live infected erythrocytes of the five other strains (data not shown). In rosette disruption assays, the antibodies to all domains except DBL2γ, when tested at 1/20 dilution of serum, abolished rosetting in R29, but had no activity against the five other strains (Figure 7). Similarly, in rosette inhibition assays at 100 µg/ml of purified IgG, the antibodies were only active against R29 (Figure 8). Thus the ITvar9 antibodies are highly effective against R29 rosetting, but show no functional cross-reactivity to other strains.

### Antibodies to ITvar9 promote phagocytosis of infected erythrocytes

**Figure 9:**
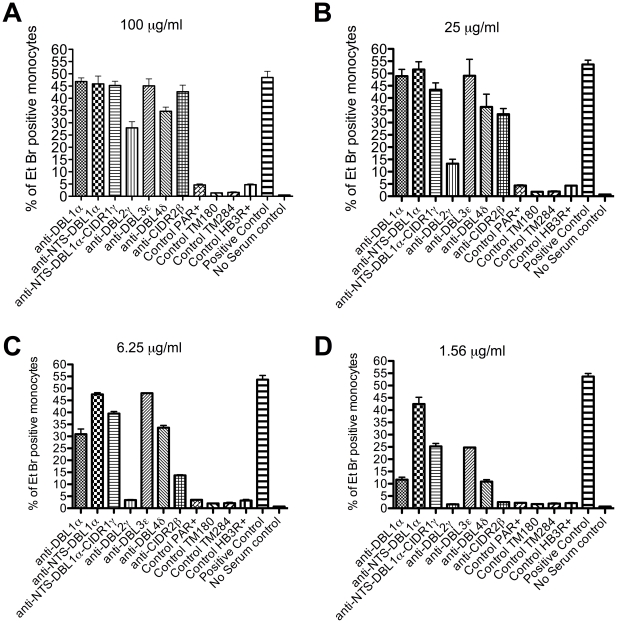
Phagocytosis of R29 infected erythrocytes after opsonization with anti-PfEMP1 antibodies. Ethidium bromide stained R29-infected erythrocytes were opsonized with antibodies and incubated with the monocytic Thp-1 cell line. The percentage of Thp-1 cells that had phagocytosed one or more infected erythrocytes was assessed by flow cytometry. The positive control was 90 µg/ml rabbit-anti human erythrocyte polyclonal antibody and the negative control was media alone (no serum control). All antibodies to PfEMP1 domains were used at four different concentrations: 100 µg/ml (A), 25 µg/ml (B), 6.25 µg/ml (C) or 1.56 µg/ml (D). Antibodies directed against ITvar9 PfEMP1 domains (first seven bars of each graph) promoted phagocytosis of R29 infected erythrocytes, whereas antibodies to the NTS-DBL1α domains of other PfEMP1 variants (control PAR+, TM284, TM180 and HB3R+) did not. The effect of ITvar9 PfEMP1 antibodies was concentration-dependent, with anti-NTS-DBL1α being the most effective at low concentration (D). Values shown are means and standard deviation from duplicates.

The ability to produce antibodies that inhibit rosette formation is desirable in order to prevent the pathological vaso-occlusive effects of rosetting. However, the ability to produce antibodies to opsonize infected erythrocytes and target them for clearance by phagocytosis would also be useful in a vaccine. We therefore investigated whether the antibodies raised to domains of ITvar9 were effective as opsonins. Fluorescently labelled R29 infected erythrocytes were preincubated with PfEMP1 antibodies over a range of concentration from 1.56–100 µg/ml and then mixed with the phagocytic cell line Thp-1 [39]. The percentage of cells that had phagocytosed at least one infected erythrocyte after 40 mins co-incubation was determined by flow cytometry. At high concentration (100 µg/ml) antibodies to all ITvar9 domains promoted phagocytosis of R29 infected erythrocytes (Figure 9A). This was a specific effect of the PfEMP1 antibodies, because in the absence of opsonizing antibodies, no phagocytosis occurred (Figure 9A, no serum control). Antibodies to NTS-DBL1α domains from other PfEMP1 variants, which do not recognize R29 live infected erythrocytes by IFA, also did not induce phagocytosis of R29 infected erythrocytes, even at high concentrations (Figure 9, control PAR+, TM180, TM284, HB3R+). At lower concentrations of ITvar9 antibodies (Figure 9B–D) there was variation in the amount of phagocytosis induced by antibodies to different domains of ITvar9, with the most efficient being the NTS-DBL1α antibodies. Consistent with the poor anti-rosetting effects of antibodies to DBL2γ (Figure 4, 7 and 8), these antibodies were the least effective at promoting phagocytosis (Figure 9).

Taken together, these results show that antibodies to the NTS-DBL1α domain were the most effective in all assays (Table 1), therefore this recombinant protein would provide the best vaccine candidate for raising antibodies against the ITvar9 PfEMP1 variant.

## Discussion

Although two previous studies have shown that antibodies to DBL1α are able to inhibit rosetting [17], [24], little is known about the effect of antibodies against the other extracellular domains of rosette-mediating PfEMP1 variants. Furthermore, whether anti-PfEMP1 antibodies have opsonizing effects has not been examined previously. In this study, recombinant domains of the ITvar9 variant were produced in *E. coli* and used to generate rabbit polyclonal antibodies for IFA, rosette disruption, rosette inhibition and phagocytosis assays. All rabbits responded well and gave antibodies that recognized the surface of R29 live infected erythrocytes, but not the surface of erythrocytes infected by other *P. falciparum* rosetting strains. As expected, antibodies to recombinant proteins containing DBL1α had anti-rosetting effects, with complete inhibition of rosetting at 1µg/ml of total IgG and an IC50 value of around 0.05 µg/ml. Unexpectedly, antibodies raised to other extracellular domains of the ITvar9 PfEMP1 variant also inhibited rosetting, with the most effective having IC50 values of around 1–5 µg/ml. Antibodies to all extracellular domains of ITvar9 were also able to opsonize infected erythrocytes for phagocytosis at high concentration. With titration, only antibodies to the different DBL1α constructs or to DBL3ε retained significant phagocytosis-inducing activity, and this was most pronounced for the NTS-DBL1α antibodies.

Much of the current work on potential PfEMP1 vaccines focuses on the generation of adhesion-blocking antibodies [28], [40], [41], [42]. Such antibodies would aim to prevent sequestration and therefore lead to clearance of mature infected erythrocytes from peripheral blood due to the filtering action of the spleen [15]. While adhesion-blocking antibodies are clearly important in preventing microvascular obstruction, it is possible that in vivo, antibodies that opsonize infected erythrocytes for phagocytosis could have equal or greater importance as a mechanism for clearance of infected erythrocytes. Previous work suggests that opsonic phagocytosis of infected erythrocytes by monocytes and neutrophils is an important mechanism of immune clearance in malaria [43], [44], [45], [46], and antibodies that recognize the surface of live infected erythrocytes have been linked to protective immunity to malaria [47], [48]. It might be expected that PfEMP1 antibodies that recognize the surface of infected cells and block adhesion would also be able to promote phagocytosis, but surprisingly, this has not previously been tested to our knowledge. Our results show that immunisation with PfEMP1 domains generates antibodies that not only block adhesion (rosetting) but also effectively opsonize and induce phagocytosis of infected erythrocytes. This dual activity would be highly advantageous in a vaccine, and it is notable that the most effective phagoctyosis inducing antibodies (to NTS-DBL1α of ITvar9) were also the most effective rosette-inhibiting antibodies (Table 1).

One of the most striking features of this study was the very high activity of the antibodies raised, especially those to NTS-DBL1α, which gave 50% inhibition of rosetting at 0.04 µg/ml of purified IgG (Figure 4B). The antibodies were equally effective with R29 parasites grown in group O erythrocytes or group A erythrocytes (which give larger, stronger rosettes, although do not alter the rosette frequency) (Figure 4D). This shows that the high activity of these antibodies is not merely a reflection of weak receptor-ligand interactions in the R29 rosetting strain in group O erythrocytes. The results shown here are not unprecedented, because rosette inhibition at low antibody concentration has been described previously for a monoclonal antibody to complement receptor 1, which inhibits rosetting at 1 and 0.1 µg/ml ([38] and JA Rowe, unpublished data). The high potency of adhesion-blocking antibodies shown here is, however, in contrast to previous studies on antibodies to DBL1α from rosetting PfEMP1 variants. One study found 50% rosette inhibition at 1/200 dilution of serum (approximately 50 µg/ml of purified IgG, assuming 10 mg/ml of IgG in normal serum) for the VarO variant in Palo Alto [17], while a second study required 1/2 dilution of serum (approximately 5 mg/ml) for the FCR3S1.2var1 variant in FCR3S1.2 parasites [24]. Several factors may contribute to the differences between studies. Firstly, the rosette inhibition assay used here is ten times more sensitive than the rosette disruption assay used in previous studies (compare Figures 4A and 4B). Secondly, it is possible that R29 rosettes are easier to inhibit than Palo Alto VarO and FCR3S1.2 rosettes, although these three strains have never been compared directly in a single laboratory. Thirdly, it is possible that the recombinant proteins used here contain more correct conformational epitopes than in previous studies, and are therefore able to induce more potent anti-rosetting antibodies. Finally, in the case of the FCR3S1.2var1 variant, in our hands this variant is expressed by non-rosetting parasites (data not shown), therefore we think it unlikely that this variant would be capable of inducing effective rosette-inhibiting antibodies.

The effectiveness of the rosette-inhibiting antibodies shown here also contrasts with current research on the pregnancy malaria vaccine candidate var2CSA. Approximately 0.5–1 mg/ml of antibodies against var2CSA single domains are needed to block infected erythrocyte adhesion to CSA [28], [42], although antibodies to the full-length protein are more effective [41]. Although var2CSA is the most promising current PfEMP1 vaccine candidate because it is relatively well-conserved across strains, the difficulty in inducing effective adhesion-blocking antibodies remains an obstacle. Our data suggest that generating adhesion-blocking antibodies against rosetting can be achieved easily, however, in this case it is the strain-specific nature of the antibodies and the between-strain variability in rosetting variants that remains the major problem.

Previous work has shown that DBL1α is the erythrocyte binding domain of rosette-mediating PfEMP1 variants [9], [17], [23]. Why then do antibodies to other extracellular domains of the ITvar9 variant inhibit rosetting? It may be that multiple domains of PfEMP1 contribute to a single binding pocket, that multiple domains interact with different receptors, or that antibodies to other domains interfere with DBL1α-receptor interactions by steric hindrance or by disrupting interactions which stabilize higher-order organization of the protein. There are some examples of PfEMP1 variants where binding to host receptors is localized to a single domain (for example, CIDRα binding CD36 [49] or DBLβC2 binding ICAM-1 [50]. However, in the case of CSA-binding, although single domains of the var2CSA PfEMP1 variant do bind CSA, the full-length molecule binds with 10,000 fold higher affinity [41], [51]. The binding affinity of full-length rosetting PfEMP1 variants compared to single domains has not yet been examined. Further work is also needed to determine if the results shown here, that multiple PfEMP1 domains can induce anti-rosetting antibodies, are replicated with other rosetting variants.

The only domain of ITvar9 that was notable for its lack of effectiveness in eliciting rosette-inhibiting and phagocytosis-inducing antibodies was DBL2γ. The reason for this is unclear, because antibodies that recognized the surface of live infected erythrocytes were generated, which were positive by IFA down to 0.39 µg/ml (Table 1). One previous study raised antibodies to the second DBL domain of a rosetting PfEMP1 variant (DBL2βC2 of VarO), and also found that the antibodies recognized the surface of live infected cells, but did not disrupt rosettes [52]. Whether this result can be generalized to all rosetting variants will require further investigation, but these initial findings do suggest that for these particular variants, DBL2 domains are not suitable vaccine candidates.

The results shown here indicate that it is straightforward to generate recombinant PfEMP1 domains in *E. coli* that induce anti-rosetting and opsonizing antibodies after immunization. This is an advance on previous work, in which generation of antibodies recognizing native PfEMP1 from *E. coli*-produced protein was problematic [17], [24], [25], [26], [27], [28], and researchers were forced to use technically more complicated and expensive expression systems such as Semliki forest virus[24] or Baculovirus [17]. More recently, Juillerat *et al* reported successful expression of a rosette-mediating PfEMP1 domain in *E. coli* as a fusion protein with maltose-binding protein, and showed that addition of the NTS to DBL1α resulted in soluble protein [52]. In our study, methods which combine structure-lead design of domain boundaries with the use of a modified pET expression vector in Origami B cells [29], reliably produced soluble recombinant PfEMP1 protein. Yields of protein were low (0.1–2 mg/l), however, it is likely that this could be improved upon by codon optimization of the expression constructs for *E. coli* if higher yields are required [53], [54].

Developing a strategy to block rosetting of *P. falciparum* could lead to a blood-stage vaccine with the potential to combat severe malaria. However, the sequence polymorphism of PfEMP1 variants, and the ability of the parasite to undergo antigenic variation and switch to different PfEMP1 variants means that targeting a single rosette-mediating variant is unlikely to be successful. An effective vaccine would either induce antibodies that cross-react with all rosetting variants, or would be composed of multiple components, each targeting a major rosetting variant type. Currently there are few data on the cross-reactivity of antibodies raised to rosette-mediating PfEMP1 variants or on the number of distinct rosetting mechanisms that occur in natural parasite populations. Here, although it was easy to generate anti-rosetting antibodies by immunizing with ITvar9 recombinant proteins, the antibodies were strain-specific and did not show functional effects on rosetting in five other laboratory strains (TM180, Muz12, TM284, HB3R+ or PAR+). Wider testing against a panel of *P. falciparum* field isolates from different malaria endemic countries will be required to determine whether ITvar9 domains induce antibodies that recognize natural parasites, and could therefore be a useful component of a multi-domain anti-rosetting vaccine. Previous work has shown that individuals from malaria endemic countries develop antibodies that recognize DBL1α of ITvar9 by ELISA [55], suggesting that epitopes within this variant are similar to those occurring commonly in natural populations. However, sera from only 3/150 Kenyan children were able to disrupt R29 rosettes, suggesting that ITvar9-like variants are rare in the Kenyan *P. falciparum* population [5]. Although the ITvar9 antibodies generated here appear to be strain-specific, this cannot be generalized across all rosetting variants without further study, and our preliminary data suggest that other rosetting PfEMP1 variants may induce cross-reactive antibodies (Ghumra and Rowe, unpublished data).

In summary, this study shows that recombinant PfEMP1 (ITvar9) domains produced in *E. coli* elicit anti-rosetting and opsonizing antibodies. NTS-DBL1α is the most promising vaccine candidate because it induces highly effective rosette-inhibiting and phagocytosis-inducing antibodies. However, several other PfEMP1 domains also generate antibodies that inhibit rosetting at slightly higher concentrations and are also effective in promoting phagocytosis. Therefore multiple extracellular domains of rosette-mediating PfEMP1 variants could be considered as vaccine candidates. Any effort to develop an effective vaccine against *P. falciparum* rosetting parasites will require recognition of multiple strains and further work is needed to fully understand the diversity of rosette-mediating variants in the field.

## Materials and Methods

### Ethics statement

Animal immunisations were carried out commercially by BioGenes GmbH (Berlin, Germany) according to European Union guidelines 86/609/EWG of 24.11.1986 and the European Agreement of 18.3.1996 for protection of animals used for scientific purposes.

### Parasites

The *P. falciparum* laboratory strains used in this study were R29, PAR+, TM284, TM180, Muz12 and HB3R+. R29 and PAR+ are rosetting clones derived from the IT/FCR3 strain, which express different PfEMP1 variants [[9] and unpublished data]. Muz12 was collected in Papua New Guinea [56], TM284 was isolated from a Thai patient with cerebral malaria and TM180 from a Thai patient with acute malaria [57]. All of the above clones/strains show high rosette frequency in culture (>50% of infected erythrocytes in rosettes), but require selection for rosetting 1–2 times per week to maintain the rosetting phenotype. Selection is carried out by centrifugation through 60% Percoll, or gelatin flotation [58]. In both cases, the rosetting parasites sediment with uninfected erythrocytes at the bottom of the tube, whereas non-rosetting infected erythrocytes are found in the top layer. HB3R+ was derived from the HB3 laboratory strain (originally from Honduras), which naturally shows a low level of rosette formation (<5%). Repeated rosette selections as described above, over the course of 3–4 weeks, resulted in the HB3R+ strain with >50% of infected erythrocytes in rosettes.

### Parasite culture

Parasites were cultured with group O erythrocytes (Scottish National Blood Transfusion Service, Edinburgh, UK) at 2% haematocrit using supplemented RPMI 1640 media as described [59]. Medium was changed daily, and fresh erythrocytes added every other day. Parasitaemia was maintained at 5–10% and cultures were incubated at 37°C in the presence of 3% CO_2_, 1% O_2_ and 96% N_2_. Cultures were screened regularly to exclude mycoplasma contamination [60].

### Assessment of rosette frequency (RF)

The rosette frequency was determined by counting a wet preparation of stained culture suspension (25µg/ml ethidium bromide for 5 mins) viewed with simultaneous white light and fluorescence as described [61]. An infected erythrocyte that bound to two or more uninfected erythrocytes was counted as a rosette. The rosette frequency is the percentage of infected erythrocytes forming rosettes out of 300 infected erythrocytes counted. Samples were blinded in all experiments to prevent observer bias.

### Expression of ITvar9 PfEMP1 domains in *E.coli*

Expression constructs were prepared for each of the six extracellular domains encoded by the *ITvar9* gene (accession codes Y13402 and CAA73831). Domain boundaries were predicted by comparing the *ITvar9* sequence with known structures of DBL [30] and CIDR [32] domains. The domain boundaries were Glu61-Pro464 for DBL1α, Met1-Pro464 for NTS-DBL1α, Met1-Val729 for NTS-DBL1α-CIDR1γ, Gln799-Thr1127 for DBL2γ, Asn1143-Gln1496 for DBL3ε, Ala1615-Pro2087 for DBL4δ and Gly2189-Leu2352 for CIDR2β. Primers were designed to amplify different fragments of *ITvar9* from IT strain genomic DNA by PCR, resulting in products with a *BamH*I cleavage site at the 5′ end and an *Nhe*I site at the 3′ end. Primers were as follows: NTSf: GGGGATCCATGACGCCAAAGCGTACAAGTC; DBL1f: CCGGATCCTCGTGTAGTCTTGATCACAAATTC; DBL1r: CCGCTAGCTTAAGGACATGCTTCACAATATC; CIDR1f: GGGGATCC

GATAATAAAATTGCATTTAATGTATTG; CIDR1r: GGGCTAGCTTATACAC

TTTTAGGTAATATATCAC; DBL2f: CTGGATCCGCGTGTGCTATTGTTAA

GGGTGTT; DBL2r: TTGCTAGCTTATGTGCACCCGCAATATTGGTC; DBL3f: CTGGATCCAATTGTTGTGGTCTAAACAGTG; DBL3r: AAGCTAGCTCATTGG

CACTCACATCTGTCTTT; DBL4f: AAGGATCCGCATGTAACCAAAAA

TATGGTTACCC; DBL4r: AAGCTAGCTCAAGGACACGGCGTACAATTTG; CIDR2f: TTGGATCCAATTGTAAAAATGGTAATTGTGGTG; CIDR2r: TTGCTAGCTCAAACACATTTTTCTTCTAGGACTGT. The PCR-amplified products were digested with restriction enzymes and inserted into a modified pEt15b vector [29], generating constructs with an N-terminal His tag and a Tobacco Etch Virus (TEV) protease cleavage site before the start of the domain [33]. Constructs were sequenced to ensure that no PCR-generated errors were incorporated.

Constructs were transformed into Origami B (Novagen, Nottingham, UK) *E. coli* containing the pRIG plasmid [33]. Cells were grown to an optical density of 1.0 at 600nm and then induced with 1mM IPTG. Expression took place at 25°C overnight. Western blots, in which the proteins were visualised using an HRP conjugated penta-His antibody (Qiagen, Crawley, UK), showed that all constructs except for CIDR1γ and DBL1α generated soluble protein. CIDR1γ produced heavily degraded protein that could not be purified while DBL1α was expressed in inclusion bodies.

### Purification of NTS-DBL1α, NTS-DBL1α-CIDR1γ, DBL3ε, DBL4δ and CIDR2β recombinant proteins

Cells were pelleted, resuspended in solubilisation buffer (20mM Tris pH 8.0, 0.3M NaCl, 10mM imidazole, 0.5% Triton X-100) and lysed by sonication. The cell lysate was centrifuged for 30 minss at 45,000g and purified by affinity chromatography using nickel-NitriloTriacetic Acid (Ni-NTA) sepharose (Qiagen). The protein was loaded onto the Ni-NTA resin, washed with solubilisation buffer, and eluted with 20mM Tris pH 8.0, 0.1M NaCl, 0.2M imidazole. Protein was buffer exchanged in 20mM phosphate pH 7.0, 150mM NaCl, 3mM reduced glutathione, 0.3 mM oxidized glutathione and cleaved overnight at room temperature by the addition of one milligram of His-tagged TEV protease [62] per ten milligrams of protein. The mixture was passed through a Ni-NTA affinity column to remove the TEV protease and any uncleaved protein that retained its His-tag. The cleaved protein in the flow-through was concentrated using an Amicon Ultra centifugal filter device (10,000 MWCO) and further purified using a Superdex 200 16/60 column (GE Healthcare, Little Chalfont, Buckinghamshire, UK) run using 20mM Tris pH 8.0, 50mM NaCl.

### Purification of DBL1α recombinant protein

The DBL1α domain was expressed as inclusion bodies and was refolded while bound to a Ni-NTA affinity column. Cells were resuspended, lysed and centrifuged as above. The pellet was resuspended by sonication in 20mM Tris pH 8.0, 0.3M NaCl, 10mM imidazole, 6M guanidine hydrochloride and 10mM β-mercaptoethanol. After clarification by centrifugation for 30 mins at 45,000g, the supernatant was loaded onto a Ni-NTA column. The column was then washed with resuspension buffer lacking β-mercaptoethanol and containing 3mM reduced glutathione and 0.3 mM oxidized glutathione. The guanidine concentration was slowly reduced from 6M to zero using a continuous gradient over 40 column volumes, while other buffer components (as described above) were maintained. The column was washed with 0.3M NaCl, 20mM Tris pH 8.0 and the protein was eluted with 20mM Tris pH 8.0, 0.1M NaCl, 0.2M imidazole. The protein was then buffer exchanged, TEV cleaved and gel filtered as above.

### SDS-PAGE of recombinant proteins

The purity of the recombinant ITvar9 DBL/CIDR proteins was assessed by SDS-PAGE on 10% bis-Tris polyacrylamide gels, stained with SimplyBlue (Invitrogen). Two micrograms of non-reduced and reduced (heated to 80°C for 10 mins in the presence of 5% β-mercaptoethanol) proteins were loaded in each well and separated by electrophoresis according to the manufacturer's protocol (Invitrogen).

### Generation of polyclonal antibodies to ITvar9 PfEMP1 domains

All immunizations were carried out by BioGenes GmbH (Berlin, Germany). Because some rabbit sera contain heterophile antibodies that react with human erythrocytes or *P. falciparum* infected erythrocytes [6], rabbits' sera were screened before immunisation using immunofluorescence assays (IFA) to select animals with minimal reactivity to parasite cultures. For each recombinant protein, pre-immune sera from five rabbits were tested for binding to R29 infected erythrocytes as described below. Two animals whose pre-immune sera gave no fluorescent signal on infected and uninfected erythrocytes were chosen for immunizations. Briefly, two rabbits were immunized with 125 µg of protein on day 0, 7, 14 and 28. Antisera from both rabbits were collected on day 28 and showed a titre of at least 1∶ 10,000 by ELISA (Biogenes GmbH). IFA on live R29 infected erythrocytes were carried out with pre-immune and post-immunization sera for both animals, and the antiserum giving the brightest positive fluorescence was chosen for Protein-A purification of total IgG (Biogenes GmbH). IgG from a non-immunised rabbit was also purified by the same method to use as a negative control, because there was insufficient pre-immune serum from the immunised rabbits to provide purified IgG for all experiments. However pre-immune sera were used (and found to be negative) in the initial immunofluorescence assays at 1/50 dilution described below, and in the rosette disruption experiments shown in Figure 7.

### Live parasite immunofluorescence assays (IFA)

IFA were carried out to i) determine if the antisera recognized native PfEMP1 on the surface of unfixed cells, ii) determine which antisera would be chosen for total IgG purification and iii) assess whether antisera cross-reacted with multiple parasite strains. Parasite cultures with a parasitaemia of at least 5% mature pigmented trophozoites and a rosette frequency of at least 50% were used. IFA were as described previously [6]. Briefly, primary incubation was for 1 hour on ice with a 1/50 dilution of pre-immune serum or anti-serum, and after three washes, secondary incubation was for 45 mins on ice with a 1∶1000 dilution of highly cross-absorbed Alexa Fluor 488-labelled goat anti-rabbit IgG (catalogue number A11034, Invitrogen Ltd, Paisley, UK) in PBS/1%BSA containing 1 µg/ml 4, 6-diamidino-2-phenylindole (DAPI). Positive IFA signals were titred out by starting with a concentration of 25 µg/ml of purified IgG, and carrying out seven 4-fold dilutions down to 0.0015 µg/ml. The end titre was the lowest antibody concentration that gave punctate fluorescence over more than 50% of infected erythrocytes. End titres were assessed by two independent observers who identified the same end point in all cases.

### Rosette disruption assays

Rosette disruption assays were carried out with parasites of greater than 50% rosette frequency. Parasite cultures were pre-stained with 25 µg/ml of ethidium bromide (5 mins at 37°C) then washed twice with incomplete RPMI 1640 (with supplements for parasite culture but without serum). The cells were resuspended at 2% haematocrit in complete binding medium (RPMI 1640 with supplements for parasite culture but lacking sodium bicarbonate), containing 10% pooled human serum that had been heat-inactivated at 56°C for 30 mins. The bicarbonate-free binding medium maintains a more stable pH in non-gassed cultures than RPMI containing bicarbonate. Rabbit pre-immune and antisera were added to 50 µl aliquots of synchronous parasite cultures (mature pigmented trophozoites, 2% haematocrit, between 5–10% parasitaemia) and incubated at 37°C for 30 mins. Negative controls were equivalent volumes of complete binding medium and serum from a non-immunised rabbit. The samples were blinded and 1.2 µl from each tube was placed on a spot of a multispot slide (Hendley Essex Ltd, Loughton, UK), and covered with a 22×22 mm coverslip. The rosette frequency was counted as described above. The data are shown as percentage of the negative control with complete binding medium added.

### Rosette inhibition assays

These assays were set up using synchronous ring-stage parasites that had shown a rosette frequency of at least 50% in the previous cycle. Parasite cultures (parasitaemia 5–10%) were spun down and resuspended in complete binding media at 2% haematocrit, and aliquoted into wells of a 96-well tissue culture plate. Total IgG, purified from antisera raised against ITvar9 recombinant proteins, was added to triplicate wells to give a final concentration of 100, 10, 0.1 and 0.01 µg/ml and a final assay volume of 100 µl. An equivalent volume of complete binding media or purified total IgG from a non-immunised rabbit was added to triplicate wells as negative controls. Tubes containing working antibody stocks diluted in complete binding media or negative controls were blinded prior to addition into the 96 well plate. The plate was put in a sealed humid chamber, supplied with a gas mixture as described above and incubated at 37°C overnight. The following day, wells were incubated with 25 µg/ml ethidium bromide for 5 mins and rosette frequency was assessed as described above. The data are shown as percentage of the negative control with complete binding medium.

### ELISA for recognition of NTS-DBL1α recombinant protein

Wells of an ELISA plate were coated with 2 µg/ml of NTS-DBL1α recombinant protein in carbonate bicarbonate buffer (Sigma, Poole, UK) and incubated overnight at 4°C. After blocking for 1 hr in PBS containing 0.05% Tween 20 (PBST) and 5% milk, wells were incubated with anti-PfEMP1 rabbit antisera diluted in PBST containing 1% milk (PBSTM). After 1 hr incubation at room temperature, wells were washed with PBST and incubated with 1∶10 000 of HRP-conjugated goat anti-rabbit IgG (Sigma) in PBSTM for another hour. Finally, wells were washed with PBST and reactions were developed by incubating the wells with substrate 3,3′,5,5′-tetramethylbenzidinedihydrochloride (Sigma) according to the manufacturer's instructions and absorbance was measured at a wavelength of 450 nm.

### Absorption of PfEMP1 antibodies on NTS-DBL1α recombinant protein coupled to sepharose

NTS-DBL1α protein was coupled to sepharose using cyanogen bromide according to the manufacturer's instructions (Sigma). Total IgG against NTS-DBL1α, NTS-DBL1α-CIDR1, DBL3ε and DBL4δ were diluted to a concentration of 1mg/ml and 100 µl of each was incubated with 100 µl of NTS-DBL1α-sepharose that had been coated with 0.3 mg of protein. Antibodies were allowed to bind for 30 mins on a rotator at room temperature. The NTS-DBL1α-sepharose was pelleted by spinning in a microfuge for 30 sec and the supernatant was collected. Immunoblotting was used to confirm that anti-NTS-DBL1α binding activity had been depleted from the absorbed antibody stocks. Doubling dilutions of recombinant NTS-DBL1α protein were made, starting at 2µg/ml. Three µl spots of each dilution were placed onto nitrocellulose (Schleicher and Schuell, Dassel, Germany). After drying, the nitrocellulose was blocked in PBST containing 5% milk for 1 hr, followed by 1 hr incubation with absorbed and unabsorbed PfEMP1 antibodies at 1/1000 dilution. The membrane was then washed in PBST and incubation with HRP-labelled goat anti-rabbit IgG (Sigma) diluted in PBSTM 1∶10,000 for another hour. Following a further wash in PBST, blots were developed using liquid diaminobenzidine (DAB) (DAKO, Ely, UK) according to the manufacturer's instructions. Rosette inhibition assays were carried out with 1∶10 dilution of normal (1mg/ml) or absorbed IgG as described above.

### Phagocytosis assays with anti-PfEMP1 antibodies

The phagocytosis assay was performed as described previously [39] with modifications. The human pro-monocytic cell line Thp-1 was maintained in RPMI 1640 (GIBCO, Mulgrave, Australia) supplemented with 10% heat-inactivated Foetal Bovine Serum (FBS), 1% penicillin-streptomycin, 2 mM L-glutamine, 25mM HEPES and 55 µM 2-Mercaptho-Ethanol (Sigma) at a density below 5×10^5^ cells/ml. During the phagocytosis assay, this medium was supplemented with 100µg/ml of Heparin (T-Hep) in order to prevent rosetting. Preliminary experiments showed that the presence of heparin did not affect phagocytosis of a positive control (erythrocytes opsonized with rabbit anti-erythrocyte surface antibodies). Synchronised R29 parasites (>70% rosetting) were washed in RPMI-HEPES medium (RPMI 1640 supplemented with 25mM HEPES, 2 mM L-glutamine, 40µg/ml gentamicin and 50µg/ml hypoxanthine) and resuspended in RPMI-HEPES containing 100µg/ml of Heparin and 0.5% BSA (R-Hep) for 15 mins. Wet smears were made to ensure that rosettes had been disrupted. Mature trophozoites-infected erythrocytes were isolated using a VarioMACS magnet [63], resulting in >80% purity. Infected erythrocytes were washed in R-Hep and resuspended at 3.3×10^7^ cells/ml in 10µg/ml of ethidium bromide solution in R-Hep. After 3 further washes, cells were resuspended at 3.3×10^7^ cells/ml in R-Hep. Thirty µl per well of this cell suspension was distributed to 96-well plates where 3.3 µl of purified rabbit IgG to ITvar9 domains or control rabbit anti-human erythrocyte antibodies (ab34858, ABCAM, Cambridge, UK) had been plated. Opsonization was allowed to occur for 60mins in the dark at RT. During this time, Thp-1 cells were washed, resuspended at 5×10^5^ cells/ml in T-Hep, and 100µl aliquots were dispensed into wells of a 96-well round-bottom plate (phagocytosis plate). After opsonization the infected erythrocytes were washed thrice with R-Hep and resuspended in 100µl of T-Hep. Two 50µl aliquots were then transferred to the phagocytosis plate and phagocytosis was allowed to occur for 40 mins at 37°C in a humidified incubator with 5% CO_2_. Phagocytosis was stopped by centrifugation at 4°C. After discarding the supernatant, the cells were resuspended at 37°C in FACS Lysing solution (BD Biosciences) according to the manufacturer's instructions. Lysis was stopped by the addition of 50 µl of cold PBS (−Ca^2+^, −Mg^2+^) 2% FBS and 0.02% NaNO_3_ (FACS Buffer). After 3 washes with FACS Buffer the cells were fixed in cold 2% Paraformaldehyde in PBS and 10,000 Thp-1 cells were acquired using a FACSCalibur flow cytometer. Only minimal agglutination of infected erythrocytes mixed with antibodies to ITvar9 occurs under the conditions of this assay (occasional 2–3 cell aggregates at 100 and 25 µg/ml), which would not be expected to have a major impact on the results.

## References

[1] Snow RW, Guerra CA, Noor AM, Myint HY, Hay SI (2005). The global distribution of clinical episodes of Plasmodium falciparum malaria.. Nature.

[2] Carlson J, Helmby H, Hill AV, Brewster D, Greenwood BM (1990). Human cerebral malaria: association with erythrocyte rosetting and lack of anti-rosetting antibodies.. Lancet.

[3] Treutiger CJ, Hedlund I, Helmby H, Carlson J, Jepson A (1992). Rosette formation in *Plasmodium falciparum* isolates and anti-rosette activity of sera from Gambians with cerebral or uncomplicated malaria.. Am J Trop Med Hyg.

[4] Ringwald P, Peyron F, Lepers JP, Rabarison P, Rakotomalala C (1993). Parasite virulence factors during falciparum malaria: rosetting, cytoadherence, and modulation of cytoadherence by cytokines.. Infect Immun.

[5] Rowe A, Obeiro J, Newbold CI, Marsh K (1995). *Plasmodium falciparum* rosetting is associated with malaria severity in Kenya.. Infect Immun.

[6] Rowe JA, Shafi J, Kai OK, Marsh K, Raza A (2002). Nonimmune IgM, but not IgG binds to the surface of *Plasmodium falciparum*-infected erythrocytes and correlates with rosetting and severe malaria.. Am J Trop Med Hyg.

[7] Rowe JA, Obiero J, Marsh K, Raza A (2002). Positive correlation between rosetting and parasitaemia in *Plasmodium falciparum* clinical isolates.. Am J Trop Med Hyg.

[8] Doumbo OK, Thera MA, Kone AK, Raza A, Tempest LJ (2009). High levels of Plasmodium falciparum rosetting in all clinical forms of severe malaria in African children.. Am J Trop Med Hyg.

[9] Rowe JA, Moulds JM, Newbold CI, Miller LH (1997). *P. falciparum* rosetting mediated by a parasite-variant erythrocyte membrane protein and complement-receptor 1.. Nature.

[10] Cockburn IA, Mackinnon MJ, O'Donnell A, Allen SJ, Moulds JM (2004). A human complement receptor 1 polymorphism that reduces *Plasmodium falciparum* rosetting confers protection against severe malaria.. Proc Natl Acad Sci U S A.

[11] Rowe JA, Handel IG, Thera MA, Deans AM, Lyke KE (2007). Blood group O protects against severe Plasmodium falciparum malaria through the mechanism of reduced rosetting.. Proc Natl Acad Sci U S A.

[12] Kaul DK, Roth EFJ, Nagel RL, Howard RJ, Handunnetti SM (1991). Rosetting of *Plasmodium falciparum*-infected red blood cells with uninfected red blood cells enhances microvascular obstruction under flow conditions.. Blood.

[13] Dondorp AM, Ince C, Charunwatthana P, Hanson J, van Kuijen A (2008). Direct in vivo assessment of microcirculatory dysfunction in severe falciparum malaria.. J Infect Dis.

[14] Kyriacou HM, Steen KE, Raza A, Arman M, Warimwe G (2007). In vitro inhibition of Plasmodium falciparum rosette formation by Curdlan sulfate.. Antimicrob Agents Chemother.

[15] Rowe JA, Claessens A, Corrigan RA, Arman M (2009). Adhesion of *Plasmodium falciparum*-infected erythrocytes to human cells: molecular mechanisms and therapeutic implications.. Expert Rev Mol Med.

[16] Kraemer SM, Smith JD (2006). A family affair: var genes, PfEMP1 binding, and malaria disease.. Curr Opin Microbiol.

[17] Vigan-Womas I, Guillotte M, Le Scanf C, Igonet S, Petres S (2008). An in vivo and in vitro model of Plasmodium falciparum rosetting and autoagglutination mediated by varO, a group A var gene encoding a frequent serotype.. Infect Immun.

[18] Chen Q, Barragan A, Fernandez V, Sundstrom A, Schlichtherle M (1998). Identification of *Plasmodium falciparum* erythrocyte membrane protein 1 (PfEMP1) as the rosetting ligand of the malaria parasite *P. falciparum*.. J Exp Med.

[19] Bull PC, Berriman M, Kyes S, Quail MA, Hall N (2005). *Plasmodium falciparum* Variant Surface Antigen Expression Patterns during Malaria.. PLoS Pathog.

[20] Kyriacou HM, Stone GN, Challis RJ, Raza A, Lyke KE (2006). Differential *var* gene transcription in *Plasmodium falciparum* isolates from patients with cerebral malaria compared to hyperparasitaemia.. Mol Biochem Parasitol.

[21] Kaestli M, Cockburn IA, Cortes A, Baea K, Rowe JA (2006). Virulence of malaria is associated with differential expression of Plasmodium falciparum var gene subgroups in a case-control study.. J Infect Dis.

[22] Warimwe GM, Keane TM, Fegan G, Musyoki JN, Newton CR (2009). Plasmodium falciparum var gene expression is modified by host immunity.. Proc Natl Acad Sci U S A.

[23] Russell C, Mercereau-Puijalon O, Le Scanf C, Steward M, Arnot DE (2005). Further definition of PfEMP-1 DBL-1alpha domains mediating rosetting adhesion of *Plasmodium falciparum*.. Mol Biochem Parasitol.

[24] Chen Q, Pettersson F, Vogt AM, Schmidt B, Ahuja S (2004). Immunization with PfEMP1-DBL1alpha generates antibodies that disrupt rosettes and protect against the sequestration of Plasmodium falciparum-infected erythrocytes.. Vaccine.

[25] Singh AP, Puri SK, Chitnis CE (2002). Antibodies raised against receptor-binding domain of Plasmodium knowlesi Duffy binding protein inhibit erythrocyte invasion.. Mol Biochem Parasitol.

[26] Oguariri RM, Mattei D, Tena-Tomas C, Uhlemann AC, Kremsner PG (2003). Recombinant Duffy binding-like-alpha domains of Plasmodium falciparum erythrocyte membrane protein 1 elicit antibodies in rats that recognize conserved epitopes.. Parasitol Res.

[27] Barfod L, Nielsen MA, Turner L, Dahlback M, Jensen AT (2006). Baculovirus-expressed constructs induce immunoglobulin G that recognizes VAR2CSA on Plasmodium falciparum-infected erythrocytes.. Infect Immun.

[28] Nielsen MA, Pinto VV, Resende M, Dahlback M, Ditlev SB (2009). Induction of adhesion-inhibitory antibodies against placental Plasmodium falciparum parasites by using single domains of VAR2CSA.. Infect Immun.

[29] Higgins MK (2008). Overproduction, purification and crystallization of a chondroitin sulfate A-binding DBL domain from a Plasmodium falciparum var2csa-encoded PfEMP1 protein.. Acta Crystallogr Sect F Struct Biol Cryst Commun.

[30] Tolia NH, Enemark EJ, Sim BK, Joshua-Tor L (2005). Structural basis for the EBA-175 erythrocyte invasion pathway of the malaria parasite Plasmodium falciparum.. Cell.

[31] Singh SK, Hora R, Belrhali H, Chitnis CE, Sharma A (2006). Structural basis for Duffy recognition by the malaria parasite Duffy-binding-like domain.. Nature.

[32] Klein MM, Gittis AG, Su HP, Makobongo MO, Moore JM (2008). The cysteine-rich interdomain region from the highly variable plasmodium falciparum erythrocyte membrane protein-1 exhibits a conserved structure.. PLoS Pathog.

[33] Baca AM, Hol WG (2000). Overcominsg codon bias: a method for high-level overexpression of Plasmodium and other AT-rich parasite genes in Escherichia coli.. Int J Parasitol.

[34] Khunrae P, Philip JM, Bull DR, Higgins MK (2009). Structural comparison of two CSPG-binding DBL domains from the VAR2CSA protein important in malaria during pregnancy.. J Mol Biol.

[35] Baruch DI, Pasloske BL, Singh HB, Bi X, Ma XC (1995). Cloning the *P. falciparum* gene encoding PfEMP1, a malarial variant antigen and adherence receptor on the surface of parasitised human erythrocytes.. Cell.

[36] Ghumra A, Semblat JP, McIntosh RS, Raza A, Rasmussen IB (2008). Identification of residues in the Cmu4 domain of polymeric IgM essential for interaction with Plasmodium falciparum erythrocyte membrane protein 1 (PfEMP1).. J Immunol.

[37] Joergensen LM, Salanti A, Dobrilovic T, Barfod L, Hassenkam T (2010). The kinetics of antibody binding to Plasmodium falciparum VAR2CSA PfEMP1 antigen and modelling of PfEMP1 antigen packing on the membrane knobs.. Malar J.

[38] Rowe JA, Rogerson SJ, Raza A, Moulds JM, Kazatchkine MD (2000). Mapping of the region of complement receptor (CR) 1 required for *Plasmodium falciparum* rosetting and demonstration of the importance of CR1 in rosetting in field isolates.. J Immunol.

[39] Ataide R, Hasang W, Wilson DW, Beeson JG, Mwapasa V (2010). Using an improved phagocytosis assay to evaluate the effect of HIV on specific antibodies to pregnancy-associated malaria.. PLoS One.

[40] Avril M, Gamain B, Lepolard C, Viaud N, Scherf A (2006). Characterization of anti-var2CSA-PfEMP1 cytoadhesion inhibitory mouse monoclonal antibodies.. Microbes Infect.

[41] Khunrae P, Dahlback M, Nielsen MA, Andersen G, Ditlev SB (2010). Full-length recombinant Plasmodium falciparum VAR2CSA binds specifically to CSPG and induces potent parasite adhesion-blocking antibodies.. J Mol Biol.

[42] Salanti A, Resende M, Ditlev SB, Pinto VV, Dahlback M (2010). Several domains from VAR2CSA can induce Plasmodium falciparum adhesion-blocking antibodies.. Malar J.

[43] Celada A, Cruchaud A, Perrin LH (1982). Opsonic activity of human immune serum on in vitro phagocytosis of *Plasmodium falciparum* infected red blood cells by monocytes.. Clin Exp Immunol.

[44] Celada A, Cruchaud A, Perrin LH (1983). Phagocytosis of *Plasmodium falciparum*-parasitized erythrocytes by human polymorphonuclear leukocytes.. J Parasitol.

[45] Mota MM, Brown KN, Holder AA, Jarra W (1998). Acute Plasmodium chabaudi chabaudi malaria infection induces antibodies which bind to the surfaces of parasitized erythrocytes and promote their phagocytosis by macrophages in vitro.. Infect Immun.

[46] Sponaas AM, Freitas do Rosario AP, Voisine C, Mastelic B, Thompson J (2009). Migrating monocytes recruited to the spleen play an important role in control of blood stage malaria.. Blood.

[47] Marsh K, Otoo L, Hayes RJ, Carson DC, Greenwood BM (1989). Antibodies to blood stage antigens of *Plasmodium falciparum* in rural Gambians and their relation to protection against infection.. Trans Roy Soc Trop Med Hyg.

[48] Bull PC, Lowe BS, Kortok M, Molyneux CS, Newbold CI (1998). Parasite antigens on the infected red cell surface are targets for naturally acquired immunity to malaria.. Nature Medicine.

[49] Robinson BA, Welch TL, Smith JD (2003). Widespread functional specialization of *Plasmodium falciparum* erythrocyte membrane protein 1 family members to bind CD36 analysed across a parasite genome.. Mol Microbiol.

[50] Howell DP, Levin EA, Springer AL, Kraemer SM, Phippard DJ (2008). Mapping a common interaction site used by Plasmodium falciparum Duffy binding-like domains to bind diverse host receptors.. Mol Microbiol.

[51] Srivastava A, Gangnard S, Round A, Dechavanne S, Juillerat A (2010). Full-length extracellular region of the var2CSA variant of PfEMP1 is required for specific, high-affinity binding to CSA.. Proc Natl Acad Sci U S A.

[52] Juillerat A, Igonet S, Vigan-Womas I, Guillotte M, Gangnard S (2010). Biochemical and biophysical characterisation of DBL1alpha1-varO, the rosetting domain of PfEMP1 from the VarO line of Plasmodium falciparum.. Mol Biochem Parasitol.

[53] Yadava A, Ockenhouse CF (2003). Effect of codon optimization on expression levels of a functionally folded malaria vaccine candidate in prokaryotic and eukaryotic expression systems.. Infect Immun.

[54] Zhou Z, Schnake P, Xiao L, Lal AA (2004). Enhanced expression of a recombinant malaria candidate vaccine in Escherichia coli by codon optimization.. Protein Expr Purif.

[55] Mayor A, Rovira-Vallbona E, Srivastava A, Sharma SK, Pati SS (2009). Functional and immunological characterization of a Duffy binding-like alpha domain from Plasmodium falciparum erythrocyte membrane protein 1 that mediates rosetting.. Infect Immun.

[56] Cox MJ, Kum DE, Tavul L, Narara A, Raiko A (1994). Dynamics of malaria parasitaemia associated with febrile illness in children from a rural area of Madang, Papua New Guinea.. Trans R Soc Trop Med Hyg.

[57] Scholander C, Treutiger CJ, Hultenby K, Wahlgren M (1996). Novel fibrillar structure confers adhesive property to malaria-infected erythrocytes.. Nature Medicine.

[58] Handunnetti SM, Gilladoga AD, van Schravendijk MR, Nakamura K, Aikawa M (1992). Purification and in vitro selection of rosette-positive (R+) and rosette-negative (R−) phenotypes of knob-positive *Plasmodium falciparum* parasites.. Am J Trop Med Hyg.

[59] Corrigan RA, Rowe JA (2010). Strain variation in early innate cytokine induction by *Plasmodium falciparum*.. Parasite Immunol.

[60] Rowe JA, Scragg IG, Kwiatkowski D, Ferguson DJP, Carucci DJ (1998). Implications of mycoplasma contaminsation in Plasmodium falciparum cultures and methods for its detection and eradication.. Mol Biochem Parasitol.

[61] Deans AM, Rowe JA (2006). Plasmodium falciparum: Rosettes do not protect merozoites from invasion-inhibitory antibodies.. Exp Parasitol.

[62] Kapust RB, Tozser J, Fox JD, Anderson DE, Cherry S (2001). Tobacco etch virus protease: mechanism of autolysis and rational design of stable mutants with wild-type catalytic proficiency.. Protein Eng.

[63] Trang DT, Huy NT, Kariu T, Tajima K, Kamei K (2004). One-step concentration of malarial parasite-infected red blood cells and removal of contaminsating white blood cells.. Malar J.

[64] Smith JD, Subramanian G, Gamain B, Baruch DI, Miller LH (2000). Classification of adhesive domains in the *Plasmodium falciparum* erythrocyte membrane protein 1 family.. Mol Biochem Parasitol.

